# Impact of test set composition on AI performance in pediatric wrist fracture detection in X-rays

**DOI:** 10.1007/s00330-025-11669-z

**Published:** 2025-05-16

**Authors:** Tristan Till, Mario Scherkl, Nikolaus Stranger, Georg Singer, Saskia Hankel, Christina Flucher, Franko Hržić, Ivan Štajduhar, Sebastian Tschauner

**Affiliations:** 1 Division of Pediatric Radiology, Department of Radiology, Medical University of Graz, Auenbruggerplatz 34, Graz, 8036 Austria; 2 Department of Pediatric and Adolescent Surgery, Medical University of Graz, Auenbruggerplatz 34, Graz, 8036 Austria; 3 Faculty of Engineering, Department of Computer Engineering, University of Rijeka, Vukovarska 58, Rijeka, 51000 Croatia

**Keywords:** Artificial intelligence, Pediatric radiology, Fracture detection, Radiographs, Test sets

## Abstract

**Objectives:**

To evaluate how different test set sampling strategies—random selection and balanced sampling—affect the performance of artificial intelligence (AI) models in pediatric wrist fracture detection using radiographs, aiming to highlight the need for standardization in test set design.

**Materials and methods:**

This retrospective study utilized the open-sourced GRAZPEDWRI-DX dataset of 6091 pediatric wrist radiographs. Two test sets, each containing 4588 images, were constructed: one using a balanced approach based on case difficulty, projection type, and fracture presence and the other a random selection. EfficientNet and YOLOv11 models were trained and validated on 18,762 radiographs and tested on both sets. Binary classification and object detection tasks were evaluated using metrics such as precision, recall, F1 score, AP50, and AP50-95. Statistical comparisons between test sets were performed using nonparametric tests.

**Results:**

Performance metrics significantly decreased in the balanced test set with more challenging cases. For example, the precision for YOLOv11 models decreased from 0.95 in the random set to 0.83 in the balanced set. Similar trends were observed for recall, accuracy, and F1 score, indicating that models trained on easy-to-recognize cases performed poorly on more complex ones. These results were consistent across all model variants tested.

**Conclusion:**

AI models for pediatric wrist fracture detection exhibit reduced performance when tested on balanced datasets containing more difficult cases, compared to randomly selected cases. This highlights the importance of constructing representative and standardized test sets that account for clinical complexity to ensure robust AI performance in real-world settings.

**Key Points:**

***Question*** *Do different sampling strategies based on samples’ complexity have an influence in deep learning models’ performance in fracture detection?*

***Findings*** *AI performance in pediatric wrist fracture detection significantly drops when tested on balanced datasets with more challenging cases, compared to randomly selected cases.*

***Clinical relevance*** *Without standardized and validated test datasets for AI that reflect clinical complexities, performance metrics may be overestimated, limiting the utility of AI in real-world settings.*

**Graphical Abstract:**

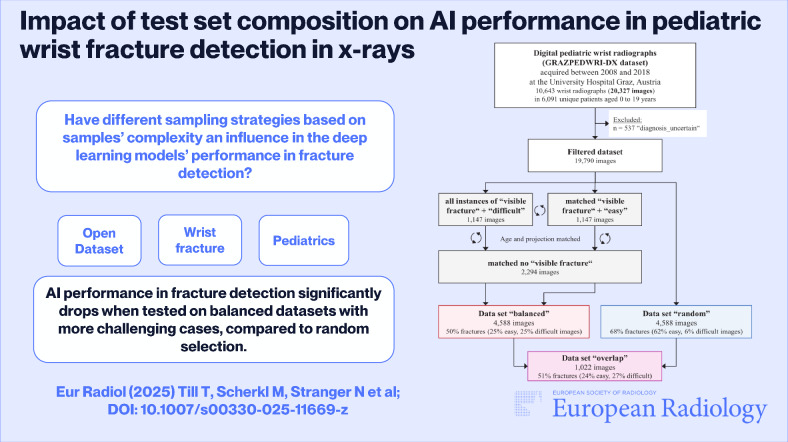

## Introduction

Artificial intelligence (AI) studies investigate the performance and accuracy of computer vision (CV) algorithms using so-called “test sets” [1]. The properties of a test set require that it is not part of the training process and initially remains unseen [2]. In mathematical terms, this means that training and test sets are disjoint sets. Ideally, the test data should come from one or more external institutions, in order to demonstrate the trained algorithms’ abilities to generalize [3, 4] on previously unseen data. The data versatility is particularly important when it comes to commercial products, certifications and regulatory approval procedures [5, 6]. Fracture detection around the wrist in digital radiographs is an increasingly important application of AI in musculoskeletal imaging and a topic of ongoing research. Several studies have been published [7–16], depicted in more detail in Table 1. Moreover, commercial products are available on the market with often reported outstanding performance values [17, 18], but when applied in clinical practice, the same performance values are hard to repeat [19]. The lack of a publicly accessible reference test set or a test set protected by a certification body stands in the way of an objective and comparable measurement of performance characteristics among different AI solutions [20].

**Table 1:** List of scientific manuscripts with regard to information on the creation and properties of the associated internal or external test sets

Study title	Authors	Year	Journal	Test set selection	Test set images (*n*)
Artificial intelligence in fracture detection: transfer learning from deep convolutional neural networks	Kim DH, MacKinnon T	2018	Clin Radiol	random	100 (balanced)
Critical evaluation of deep neural networks for wrist fracture detection	Raisuddin, AM, Vaatto-vaara, E et al	2021	Scientific Reports	random-difficult	414-210
Assessment of an AI Aid in Detection of Adult Appendicular Skeletal Fractures by Emergency Physicians and Radiologists: A Multicenter Cross-sectional Diagnostic Study	Duron L, Ducarouge A, Gillibert A et al	2021	Radiology	consecutive	600
Artificial intelligence for diagnosing distal radius fractures using biplane plain X-rays	Oka K, Shiode R, Yoshii Y et al	2021	J Orthop Surg Res	random	120
Improving Radiographic Fracture Recognition Performance and Efficiency Using Artificial Intelligence	Guermazi A et al	2022	Radiology	random	480
Pediatric radius torus fractures in x-rays—how computer vision could render lateral projections obsolete	Janisch M, Apfaltrer G, Hržić F et al	2022	Front. Pediatr	random	200 (balanced)
Commercially available AI algorithm improves radiologists’ sensitivity for wrist and hand fracture detection on X-ray, compared to a CT-based ground truth	Jacques T, Cardot N, Ventre J et al	2023	Eur Radiol	CT patients	788
Artificial intelligence vs. radiologist: accuracy of wrist fracture detection on radiographs	Cohen M, Puntonet J, Sanchez J et al	2023	Eur Radiol	consecutive	1917 patients
Detecting pediatric wrist fractures using deep-learning-based object detection	Zech JR, Carotenuto G, Igbinoba Z et al	2023	Pediatr Radiol	random	125
Fracture detection in pediatric wrist trauma X-ray images using YOLOv8 algorithm	Ju R-Y and Cai W	2023	Scientific Reports	random	2029

Here, *random* denotes a fully random test-selection; *random-difficult* a random selection of data, for which the difficulty of cases was taken into consideration during testing, while *consecutive* proposes a setting, in which chronologically consecutive cases are used for testing, as to simulate an application environment. Note the substantial heterogeneity of reported test set samples

Another important aspect is the “difficulty” of the cases tested [15]. To be precise, when sampling data for the training, validation and test sets, is it justified to treat each data sample as equally demanding and important? The majority of previous studies shown in Table 1 were satisfied with randomly selecting test set cases or analyzing consecutive case series. However, different allocation strategies influence this selection of samples and consequently yield a significant impact on the resulting performance metrics [21, 22]. In the studied case of fractures, easily recognizable fractures are not a challenge for experienced investigators, and in a clinical environment, algorithms detecting only easy fractures are not of great help.

The hypothesis of this study was that different sampling strategies based on samples’ complexity significantly influence the deep-learning models’ performance. To test the set hypothesis, two test sets were separated from the dataset, one with a balanced distribution of features, compared to one with a random distribution. Afterwards, two standard computer vision (CV) algorithms, which were trained on the same training and validation data, were tested on these test sets. The difference in predictive performance is reported here.

## Materials and methods

### Dataset

In this retrospective study, the open-sourced GRAZPEDWRI-DX dataset [23] containing annotated radiographs of pediatric wrist fractures of 6091 patients was first supplemented with additional information on the difficulty of the case. A pediatric radiologist with 11 years of experience in musculoskeletal radiology categorized the images into easy and difficult groups by subjectively grading the visibility of fractures. These subjective ratings were stored in a separate column of the datasets meta information.

Furthermore, input images were preprocessed by using scikit-image package version 0.19. We performed a percentile crop with 2% at the lower end and 0.5% at the upper range of gray values. Afterwards, a local contrast enhancement (CLAHE) was applied to ensure proper contrast in the images. The PNG files were then converted to RGB color-images with 8 Bits per channel, as standard input for neural networks.

#### Dataset splits

**Fig. 1:**
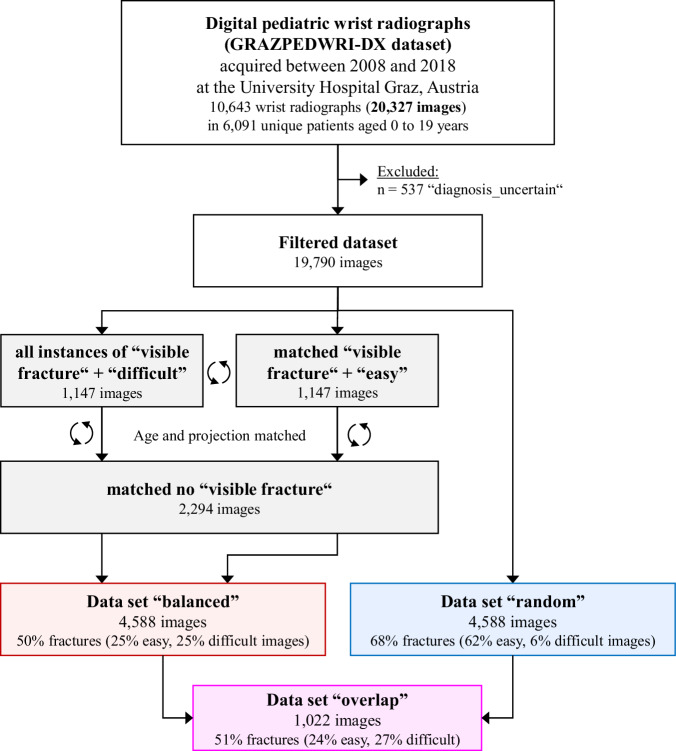
Flowchart of the dataset [23], including subsamples, splits and matching procedures to generate the datasets ”balanced”, ”random” and ”overlap”

Due to the finite number of images available for neural network training, validation, and testing, we created two subsets for later comparison, each containing 4588 samples (compare Fig. 1).

- **“Balanced”**: The first dataset was composed of X-rays that were balanced in the parameters “difficulty”, “projection”, and “fracture”. This was accomplished using a Python script and a custom matching algorithm. The first step was to exclude images labeled with the “diagnosis uncertain” tag. The remaining 1147 difficult cases with visible fractures were then matched to easy images based on “age” and “projection”. Subsequently, the 2294 fracture cases were matched again to images without visible fractures. This dataset contained 4588 images total, with 50% fractures and half of the fractures being difficult samples.
- **“Random”**: A random selection of 4588 images from the entire dataset served as a control group. This “Random” test set had the same distribution of properties as the whole GRAZPEDWRI-DX dataset, without images flagged “diagnosis uncertain”. The properties are similar to a consecutive series of patients.
- **“Overlap”**: The dataset splits overlapped in 1,022 images due to the individual sampling procedures, specifically in 691 easy and 331 difficult X-rays. Performance metrics in this subset were also evaluated.

Patient characteristics were comparable in the “balanced” and “random” datasets Table 2. The distribution of gender, laterality, and initial examination did not show relevant differences between the test sets. Note that the rate of difficult cases was 6% in the “random” subset, but 25% (50% of the images featuring a fracture, *n* = 1147) in the “balanced” subset.

**Table 2:** Patient characteristics and case parameters for test sets and the training set

Parameter		Dataset 1		Dataset 2		Dataset 3	
		“balanced”		“random”		“overlap”	
		*n* = 4588		*n* = 4588		*n* = 1022	
Age	Mean (years)	11.73		10.9		10.74	
	SD (years)	3.32		3.58		3.28	
	Min (years)	0.40		0.40		0.40	
	Max (years)	18.00		18.70		18.00	
Gender	Female	1981	43%	1930	42%	454	44%
	Male	2607	57%	2658	58%	568	46%
Laterality	Left	2388	52%	2592	56%	534	52%
	Right	2200	48%	1996	44%	488	48%
Projection	a.p.	2504	55%	2216	48%	542	53%
	Lateral	2080	45%	2344	52%	478	47%
	Oblique	4	0%	28	0%	2	0%
Study type	Initial	1482	32%	996	22%	775	76%
	Follow-up	3106	68%	3592	78%	247	24%
Fracture	Yes	2294	50%	3132	68%	527	52%
	No	2294	50%	1456	32%	495	48%
Difficulty (in fractures)	Easy	1147	25%	2860	62%	254	25%
	Difficult	1147	25%	273	6%	273	27%

Parameters, which can occur more than once, are counted on a per-image, not per-case, basis

### Model training

The set hypothesis was evaluated on two separate tasks. The first task (task 1) was to perform binary classification of fracture presence in the X-ray, while the second task was to detect fractures and mark the fracture region (object detection, task 2). In the task of binary classification, radiographs annotated with fracture bounding boxes were considered positive for the presence of a fracture even if occult fractures had been identified in other projections of the same examination or from the course of treatment.

All models were trained on a Linux workstation equipped with two Nvidia RTX 4090 graphics cards with 24 GB of video memory each. The CUDA 12.4 framework was used. The CPU was an Intel Core i7 13700 K, and 64 GB of RAM was available. We used Python 3.10 on an Ubuntu 22.04 LTS build.

A 10-fold cross-validation method was computed for both tasks with the aim to efficiently use all the available image samples. This means that all trained samples were also available for performance metrics calculations. A selection of neural network settings is given in Table 3.

**Table 3:** Neural network configurations for YOLOv11 and EfficientNet

Neural network	Input size	Batch size	Dataloaders	Epochs	K-fold splits	Train-val split
	(pixels)	(*n*)	(*n*)		(*n*)	(%)
YOLOv11n	640	128	2	100	10	80/20
YOLOv11s	640	84	2	100	10	80/20
YOLOv11m	640	48	2	100	10	80/20
YOLOv11l	640	32	2	100	10	80/20
YOLOv11x	640	16	2	100	10	80/20
EfficientNet-B0	224	160	8	100	10	80/20
EfficientNet-B1	240	96	8	100	10	80/20
EfficientNet-B2	260	64	8	100	10	80/20
EfficientNet-B3	300	32	8	100	10	80/20
EfficientNet-B4	380	16	4	100	10	80/20
EfficientNet-B5	456	12	4	100	10	80/20
EfficientNet-B6	528	8	4	100	10	80/20
EfficientNet-B7	600	4	4	100	10	80/20

#### Task 1: Fracture classification

For the binary fracture classification task, the EfficientNet family of models was chosen based on its success in various tasks related to medicine [24–26]. To prove the robustness of the experiment, all variations of EfficentNet (from B0 to B7) were tested, eliminating any potential bias in image resolution. All model variants of the EfficientNet were trained on their default image size (B0: 224 × 224 px, B1: 240 × 240 px, B2: 260 × 260 px, B3: 300 × 300 px, B4: 380 × 380 px, B5: 456 × 456 px, B6: 528 × 528 px, B7: 600 × 600 px) [27]. For EfficientNet training, FastAI Python packed v2.7.15 [28] was utilized. The following augmentation parameters were used in the “aug transforms” function during EfficientNet trainings: aug transforms (mult = 1, do flip = True, flip vert = True, max-rotate = 20, min zoom = 0.8, max zoom = 1.2, max lighting = 0.8, max warp = 0.2, p affine = 0.8, p lighting = 1, size = None, mode = “bilinear”, pad mode = “reflection”, align corners = False, batch = False). 20% of the samples were randomly allocated to the validation for each fold while training.

#### Task 2: Fracture localization

For the fracture localization (object detection) task for the only relevant class “fracture” the model of choice was YOLOv11 based on its success in multiple papers published on the used dataset [8, 11, 12, 29]. As model optimization was not a goal of this paper, standard settings and hyperparameters (AdamW optimizer, lr0 = 0.01, lrf = 0.01, momentum = 0.937, weight decay = 0.0005, warmup epochs = 3.0, warmup momentum=0.8, warmup bias lr = 0.1) and augmentations (cls = 0.5, dfl = 1.5, pose = 12.0, kobj= 1.0, nbs =64, hsv *h* = 0.015, hsv *s* = 0.7, hsv *v* = 0.4, degrees = 0.0, translate = 0.1, scale = 0.5, shear = 0.0, perspective = 0.0, flipud = 0.0, fliplr = 0.5, bgr = 0.0, mosaic = 1.0, mixup = 0.0, copy paste = 0.0, copy paste mode = flip, auto augment=randaugment, erasing = 0.4, crop fraction = 1.0) were used as seen in the YOLOv11 documentation [30]. The training of YOLOv11 was done by using the ultralytics package 8.3.70 [31]. Batch sizes were adapted to fit into the video memory of the graphics cards. We trained all model variants of the YOLOv11 architecture (YOLOv11n, YOLOv11s, YOLOv11m, YOLOv11l, and YOLOv11x) for 100 epochs with pre-trained weights and an input size of 640 × 640 px.

### AI performance metrics

Testing was performed in a two-step process, initially computing the average precision (AP), AP50 and AP50-95 [31], as standard performance indicators [32] for each test set. In addition, the aforementioned test sets were subdivided into their easy and difficult cases, for which the testing process was repeated as described above.

Image classification and regression performance were measured by assessing True Positives (TP): The model predicted a label that correctly matches the ground truth.

True Negatives (TN): The model correctly predicts the absence of a label, which is not present in the ground truth.

False Positives (FP): The model predicted a label, but it does not match the ground truth (Type I Error).

False Negatives (FN): The model does not predict a label, but it is present in the ground truth (Type II Error).

Precision (P): The ratio of TP to TP + FP, indicating the proportion of correct positive predictions out of all positive predictions made by the model.

Recall (R): The ratio of TP to TP + FN, measuring the model’s ability to correctly identify all relevant instances in the ground truth.

Accuracy (A): The proportion of TP + TN among the total number of cases examined, providing an overall measure of model performance.

F1 score (F1): The harmonic mean of precision and recall, providing a balanced measure that accounts for both false positives and false negatives.

Average Precision (AP): The area under the precision-recall curve, summarizing the precision-recall trade-off across different thresholds and providing a single measure of model performance.

For binary classification tasks with EfficientNet, “Precision”, “Recall”, “F1 score” and “Accuracy” were used as the primary performance metrics.

For regression and classification tasks using YOLOv11 variants, “Precision”, “Recall”, “F1 score” and “Average Precision” (AP50 and AP50-95) were used as the main measures of performance. For AP50, a TP was registered at an overlap of at least 50% between the prediction and ground truth. For AP50-95, the AP is calculated by varying the Intersection over Union (IoU) threshold from 50% to 95%, in increments of 5%, to assess the model’s performance across different levels of overlap [33].

### Statistical analyses

Statistical analyses were performed with SPSS version 27 (IBM). We used descriptive statistics and comparisons of means across the different AI model variations. Due to the restricted number of data points, we used nonparametric *Sign*-tests to compare the differences between the “Balanced” and “Random” test sets. Area under the curve (AUC) plots were generated to visualize object detection metrics. *p*-values below 0.05 were assumed to be statistically significant.

### Ethical committee

All experiments were performed according to the declaration of Helsinki. The evaluations were carried out after acquirement of a positive vote by the  Ethics Committee of the Medical University of Graz (IRB00002556), EK Number. The requirement for written informed consent was waived due to the retrospective study design.

## Results

### Task 1: Fracture classification

**Fig. 2:**
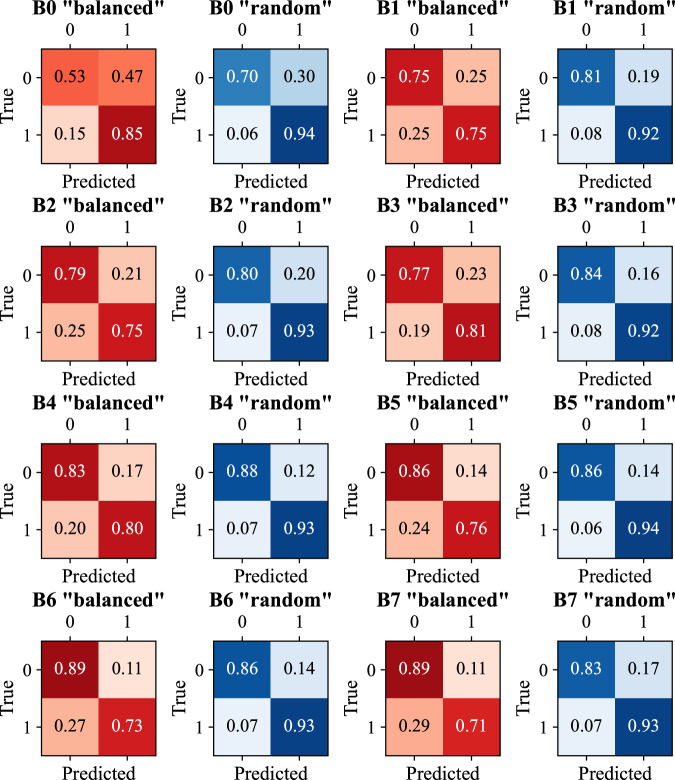
Confusion matrices of EfficientNet predictions vs. the ground truth of the presence of fractures. Matrices are given for “balanced” and “random” subsets. The numbers are normalized between 0 and 1 across all EfficientNet variants (B0 to B7)

Fracture classification metrics were significantly better in the “random” dataset compared among all 8 EfficientNet variants, with a weighted precision of 0.785 ± 0.036 and 0.894 ± 0.016, weighted recall of 0.780 ± 0.040 and 0.894 ± 0.016, and weighted F1 score of 0.778 ± 0.042 and 0.894 ± 0.017 (“balanced” vs. “random” datasets, *Sign test*, all *p* = 0.008). For both subsets, confusion matrices were calculated as shown in Fig. 2.

**Fig. 3:**
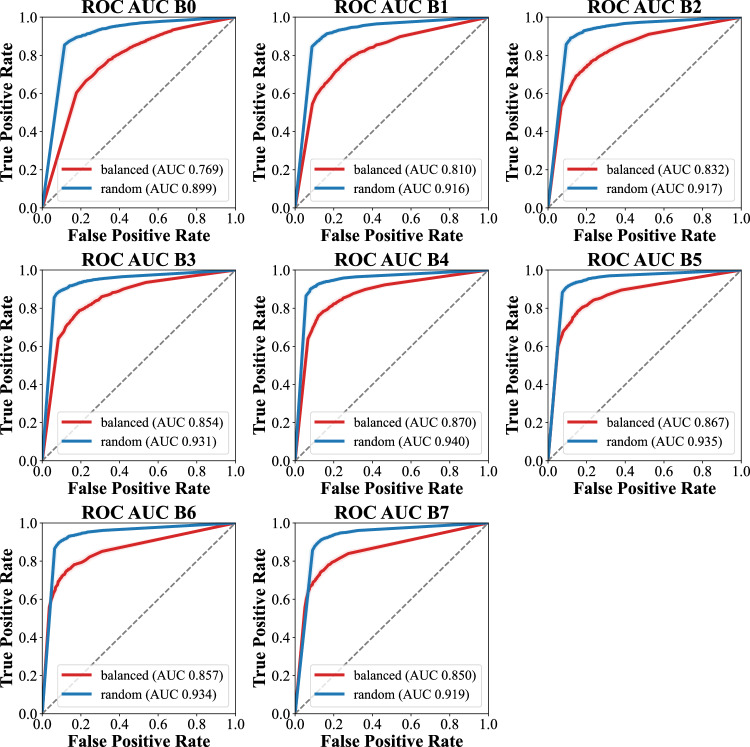
Scatter plots of EfficientNet metrics for both test sets, “balanced” and “random”

ROC analyses consistently demonstrated superior fracture classification performance in the “random” subset (*Sign test* *p* = 0.008), ranging from an AUC of 0.899 (95% CI 0.889 to 0.909) in EfficientNet-B0 to a maximum of an AUC of 0.940 (95% CI 0.931 to 0.947) in EfficientNet-B4. The AUC values in the “balanced” subset ranged from 0.769 (95% CI 0.756 to 0.782) in EfficientNet-B0 to a maximum of an AUC of 0.870 (95% CI 0.859 to 0.880) in EfficientNet-B4. The respective plots are given in Fig. 3. The charts also suggested that larger model variants, which are augmented in terms of image size, number of parameters and number of gradients, and model performance were loosely correlated regardless of the test set.

**Fig. 4:**
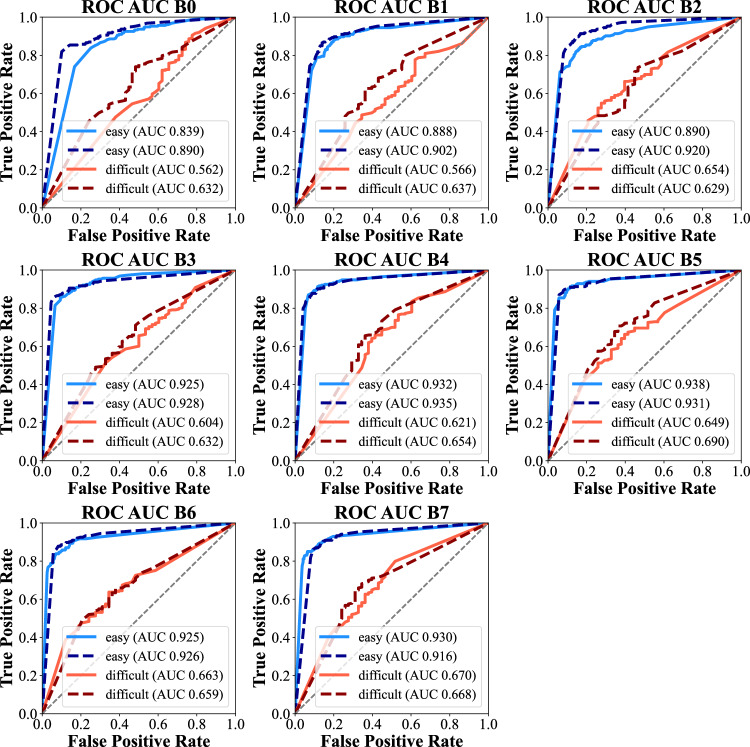
ROC analysis of the “overlap” subset for combined EfficientNet variants B0 to B7 of “balanced” = dashed line graphs and “random” = full line graphs for easy (blue, *n* = 247), difficult (red, *n* = 280) and samples without fracture (*n* = 495). AUC values were comparable between the datasets, without relevant advantages of the “balanced” subset featuring more difficult samples during training and validation

The “overlap” subset demonstrated correlating behavior without relevant variability, but generally with lower ROC AUC values (compare Fig. 3 and Fig. 4). The mean precision across the EfficientNet variants for difficult cases was 0.760 ± 0.019 (subset “balanced”) and 0.779 ± 0.011 (subset “random”, *Sign test* *p* = 0.070), and the recall was 0.631 ± 0.033 and 0.623 ± 0.031 (*Sign test* *p* = 0.727). The mean F1 score was 0.671 ± 0.025 for difficult cases in the “balanced” subset and 0.668 ± 0.026 in the “random subset (*Sign test* *p* = 1.000). The findings are illustrated graphically in Fig. 4, indicating that additional difficult training samples did not yield significant performance increases in the difficult cases. This also indicates that difficult cases are harder to learn and predict.

### Task 2: Fracture localization

**Fig. 5:**
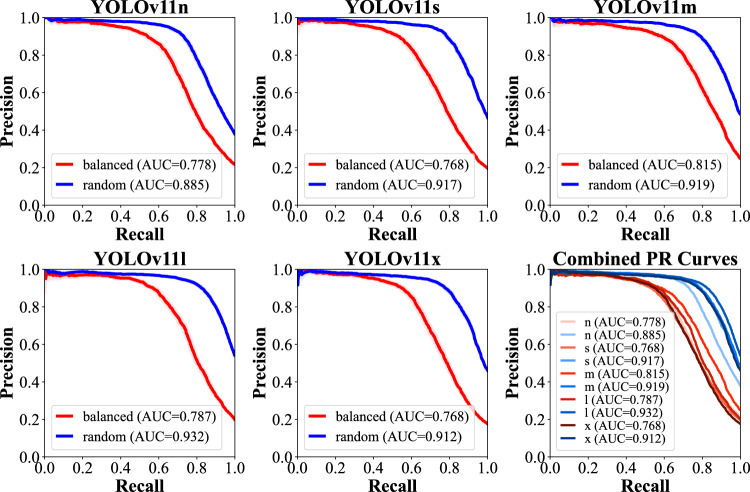
Precision-Recall curves for YOLOv11 models “n”, “s”, “m”, “l”, and “x”, compared between “balanced” (red) and “random” (blue) datasets

**Fig. 6:**
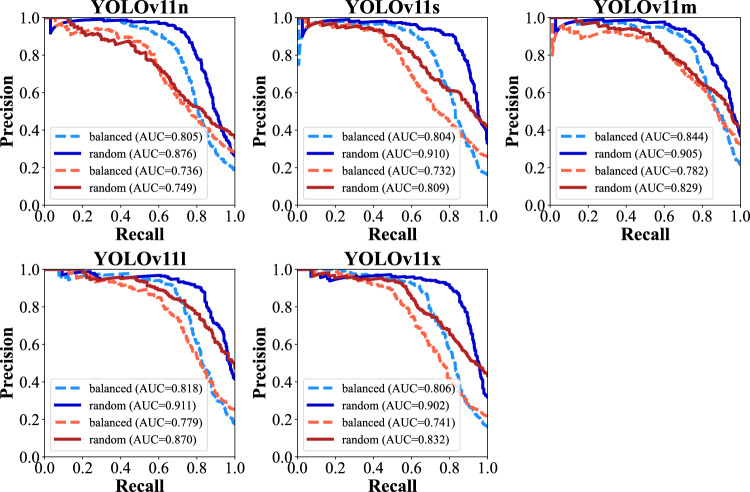
Precision-Recall curves of the “overlap” set for YOLOv11 models “n”, “s”, “m”, “l”, and “x”, compared between “balanced” (dashed line) and “random” (solid line) training data. The results for the difficult cases (red) were comparable between the “balanced” and “random” training sets. There was a drop in performance in the easy cases of the “balanced” compared to the “random” subset. This could be explained by the lower number of easy samples in the “balanced” subset

Fracture localization using the YOLOv11 AI model architecture demonstrated significantly lower performance metrics in the “balanced” subset. Precision ranged from 0.829 ± 0.032 in YOLOv11m to 0.860 ± 0.036 in YOLOv11l. Recall was lowest in YOLOv11x with 0.714 ± 0.040 and highest in YOLOv11s with 0.743 ± 0.028. AP50 was the lowest in YOLOv11x with 0.806 ± 0.025 and highest in YOLOv11s with 0.822 ± 0.014. In the “random” subset, precision ranged from 0.893 ± 0.023 in YOLOv11m to 0.910 ± 0.022 in YOLOv11s, recall ranged from 0.819 ± 0.024 in YOLOv11n to 0.832 ± 0.017 in YOLOv11s, and AP50 from 0.894 ± 0.008 in YOLOv11n to 0.903 ± 0.015 in YOLOv11s. *Sign tests* across the five model variants comparing the subsets showed a statistically significant difference for precision, recall, and AP50 (each *p* = 0.025). The performance of the YOLOv11 models in terms of PR AUC analyses is shown in Figs. 5 and 6.

The “overlap” subset was split into easy and difficult cases. In contrast to the fracture classification (task 1), model training influenced YOLOv11 performance metrics substantially. The whole fracture localization performance was substantially impaired in the “balanced” subset, not only for the difficult cases, but also for the easy cases. PR AUC values ranged from 0.732 in the difficult cases of the “balanced” subset to 0.911 in the easy cases of the “random” subset. The PR AUC metrics in difficult cases were significantly higher in the “random” subset (*Sign test*, *p* = 0.025). Figure 6 shows the findings graphically.

## Discussion

This study reports on the performance characteristics of test set variations in AI-assisted automated detection of wrist fractures in children and adolescents. Two test sets were separated from a publicly available dataset and compared with each other through variations of current convolutional neural networks for classification and object detection. One test set featured balanced patient and pathology characteristics, considering the difficulty of the fracture cases, while another test set was sampled randomly. AI prediction performance decreased substantially in challenging cases.

The novelty of this work relates to the systematic side-by-side comparison of a balanced test set (presence of fractures, difficulty, projection) and a random test set for determining the AI performance values. In contrast to Raissudin et al [15], test sets of equal size with 4588 images each were used, and the focus was not primarily on aspects of difficult cases. Moreover, a publicly available pediatric trauma x-ray dataset was used, which increases the reproducibility and transparency of our results. In routine clinical practice, the radiologist encounters all degrees of challenges in fracture detection, each of which should also be mastered by the AI. The work underlines the continuing issues regarding the performance of AI algorithms in challenging cases. The relevant publications on automated wrist fracture detection suggest that the technology is of great benefit to patients and specialist staff. The performance of AI algorithms for wrist fracture detection is commonly reported on randomly sampled cases, separated from an existing dataset [12]. Other studies measure performance on a series of consecutive patients, whereby the criteria for referral to an X-ray examination remain unclear [14]. It should be noted that the majority of patient visits represent clear situations with obvious pathologies or clearly negative findings, potentially positively skewing the performance data. Only a minority of cases are actually challenging. In terms of the dataset used, difficult cases were only 6% of all cases. This means that performance data is often determined and reported using relatively simple samples, and the excellent metrics cannot be replicated on challenging cases. There is definitely not enough research tackling the challenges of AI-based automated fracture detection, and only a few manuscripts have elaborated on the topic itself or the broader subject area before [15, 34].

Overall, the two subsets, “balanced” and “random”, showed similar behavior in terms of the fracture detection (tasks 1 and 2) rate, with consistently poorer performance of the AI models in the “balanced” variant. However, this was not the case when considering the easy and difficult cases in the “overlap” subset (*n* = 1.022 images). While the fracture classification with EfficientNet delivered almost equal results between the “balanced” and “random” subsets, the fracture localization performance using YOLOv11 suffered noticeably in the “balanced” variant. As a result, YOLOv11 variants, apart from the “n” version, performed significantly worse for easy and difficult images in the “balanced” subset. This implies that YOLOv11 might need more training samples than EfficientNet. Generally, more fracture cases were sampled in the “random” subset (68% vs. 50% fractures, compare Table 2). In any case, the results of the experiments suggest that challenging cases are indeed more difficult for image recognition algorithms to assess and that the differences are not primarily due to training bias. Humans identify objects by feature-based recognition image-specific factors [35].

Therefore, object detectors and humans perform better in easy cases because the utilized discriminatory features to compare an object in the cropped region against a target are bigger [35]. Thus, our results are in line with the currently available literature considering AI fracture prediction performance.

From the present perspective, the availability of an independent, reliable and incorruptible entity that maintains a representative test dataset for the measurement and comparability of AI algorithms for fracture detection is not foreseeable. The scientific community and specialist societies should work on providing such reference test sets as a benchmark for various radiological questions. We are in need of an “AI crash test” like in the automotive industry, where an uninvolved third party independently and objectively evaluates and provides the performance data. As long as no such test sets are available, the results of scientific studies must be viewed with a certain degree of skepticism, even if the studies were actually planned and carried out properly.

A limitation of our study is that it was conducted on a specific dataset, only containing pediatric patients and radiographs around the wrist. Other body regions, age groups, or data from different institutions may lead to different results. A further limitation is that only one reviewer categorized the images into easy and difficult groups by subjectively grading the visibility of fractures, since there is currently no objective grading standard. Even compared to gold standards like CT or MRI might not fully account for this drawback. Furthermore, it needs to be considered that only the fracture instances were rated in regard to difficulty. In real-world scenarios, there is also the situation that studies are often interpreted false-positively, especially in children due to the open growth plates. These cases would also warrant being categorized as difficult. A perfect test set would also need to consider the difficulty of all cases based on extensive multi-user readings. Finally, our study also was not aimed to examine generalization of AI algorithms.

As there are no standardized and validated test datasets for AI applications in (pediatric) fracture detection, performance is commonly assessed using consecutive patient series. Our experiments demonstrate that this leads to significantly better metrics than would be the case with a test set as balanced as possible. In challenging cases, where the benefits of computer assistance would be most beneficial, the performance in AI fracture detection dropped substantially, which was not due to training bias but to the increased level of difficulty. This also raises the question, which bodies or entities could potentially build and maintain reference test datasets to assess AI performance, identify problems and obtain official certifications.

## Abbreviations

AI: Artificial intelligence
AP: Average precision
AUC: Area under the curve
F1: F1 score
FN: False negatives
FP: False positives
P: Precision
R: Recall
TN: True negatives
TP: True positives

## References

[1] Kempen EJ, Post M, Mannil M et al (2021) Performance of machine learning algorithms for glioma segmentation of brain MRI: a systematic literature review and metaanalysis. Eur Radiol 31:9638–9653. 10.1007/s00330-021-08035-0 34019128 10.1007/s00330-021-08035-0 PMC8589805

[2] Freeman K, Geppert J, Stinton C et al (2021) Use of artificial intelligence for image analysis in breast cancer screening programmes: systematic review of test accuracy. BMJ 1872. 10.1136/bmj.n1872 10.1136/bmj.n1872 PMC8409323 34470740

[3] Bluemke DA, Moy L, Bredella MA et al (2020) Assessing radiology research on artificial intelligence: a brief guide for authors, reviewers, and readers—from the radiology editorial board. Radiology 294:487–489. 10.1148/radiol.2019192515 31891322 10.1148/radiol.2019192515

[4] Armato SG, Drukker K, Hadjiiski L (2023) AI in medical imaging grand challenges: translation from competition to research benefit and patient care. Br J Radiol 96. 10.1259/bjr.20221152 10.1259/bjr.20221152 PMC10546459 37698542

[5] US Food and Drug Administration et al (2024) Artificial intelligence & medical products: how CBER, CDER, CDRH, and OCP are Working Together. US Food and Drug Administration

[6] European Medicines Agency (2023) Reflection paper on the use of artificial intelligence (AI) in the medicinal product lifecycle. Draft Reflection Paper, EMA/CHMP/CVMP/83833/2023. https://www.ema.europa.eu

[7] Guermazi A, Tannoury C, Kompel AJ et al (2022) Improving radiographic fracture recognition performance and efficiency using artificial intelligence. Radiology 302:627–636. 10.1148/radiol.210937 34931859 10.1148/radiol.210937

[8] Nguyen T, Maarek R, Hermann A-L et al (2022) Assessment of an artificial intelligence aid for the detection of appendicular skeletal fractures in children and young adults by senior and junior radiologists. Pediatr Radiol 52:2215–2226. 10.1007/s00247-02205496-3 36169667 10.1007/s00247-022-05496-3

[9] Gasmi I, Calinghen A, Parienti J-J, Belloy F, Fohlen A, Pelage J-P (2023) Comparison of diagnostic performance of a deep learning algorithm, emergency physicians, junior radiologists and senior radiologists in the detection of appendicular fractures in children. Pediatr Radiol 53:1675–1684. 10.1007/s00247-023-05621-w 36877239 10.1007/s00247-023-05621-w

[10] Jacques T, Cardot N, Ventre J, Demondion X, Cotten A (2023) Commercially available AI algorithm improves radiologists’ sensitivity for wrist and hand fracture detection on X-ray, compared to a ct-based ground truth. Eur Radiol. 10.1007/s00330-023-10380-1 10.1007/s00330-023-10380-1 37919408

[11] Till T, Tschauner S, Singer G, Lichtenegger K, Till H (2023) Development and optimization of AI algorithms for wrist fracture detection in children using a freely available dataset. Front Pediatr 11. 10.3389/fped. 2023.1291804 10.3389/fped.2023.1291804 PMC10768054 38188914

[12] Ju R-Y, Cai W (2023) Fracture detection in pediatric wrist trauma X-ray images using YOLOv8 algorithm. Sci Rep 13. 10.1038/s41598-023-47460-7 10.1038/s41598-023-47460-7 PMC10654405 37973984

[13] Janisch M, Apfaltrer G, Hržić F et al (2022) Pediatric radius torus fractures in x-rays—how computer vision could render lateral projections obsolete. Front Pediatr 10. 10.3389/fped.2022.1005099 10.3389/fped.2022.1005099 PMC9794847 36589159

[14] Oka K, Shiode R, Yoshii Y, Tanaka H, Iwahashi T, Murase T (2021) Artificial intelligence to diagnosis distal radius fracture using biplane plain X-rays. J Orthopaed Surg Res 16. 10.1186/s13018-021-02845-0 10.1186/s13018-021-02845-0 PMC8620959 34823550

[15] Raisuddin AM, Vaattovaara E, Nevalainen M et al (2021) Critical evaluation of deep neural networks for wrist fracture detection. Sci Rep 11. 10.1038/s41598-021-85570-2 10.1038/s41598-021-85570-2 PMC7971048 33727668

[16] Hržić F, Tschauner S, Sorantin E, Štajduhar I (2022) Fracture recognition in paediatric wrist radiographs: An object detection approach. Mathematics 10:2939. 10.3390/math10162939

[17] Bousson V, Attané G, Benoist N et al (2023) Artificial intelligence for detecting acute fractures in patients admitted to an emergency department: Real-life performance of three commercial algorithms. Acad Radiol 30:2118–2139. 10.1016/j.acra.2023.06.016 37468377 10.1016/j.acra.2023.06.016

[18] Fu T, Viswanathan V, Attia A et al (2024) Assessing the potential of a deep learning tool to improve fracture detection by radiologists and emergency physicians on extremity radiographs. Acad Radiol 31:1989–1999. 10.1016/j.acra.2023.10.042 37993303 10.1016/j.acra.2023.10.042

[19] Nwanosike EM, Conway BR, Merchant HA, Hasan SS (2022) Potential applications and performance of machine learning techniques and algorithms in clinical practice: a systematic review. Int J Med Inform 159:104679. 10.1016/j.ijmedinf.2021.104679 34990939 10.1016/j.ijmedinf.2021.104679

[20] Fung BCM, Wang K, Chen R, Yu PS (2010) Privacy-preserving data publishing: a survey of recent developments. ACM Comput Surv 42:1–53. 10.1145/1749603.1749605

[21] Vabalas A, Gowen E, Poliakoff E, Casson AJ (2019) Machine learning algorithm validation with a limited sample size. PLoS One 14:1–20. 10.1371/journal.pone.0224365 10.1371/journal.pone.0224365 PMC6837442 31697686

[22] Pecher B, Srba I, Bielikova M, Vanschoren J (2024) Automatic combination of sample selection strategies for few-shot learning. http://arxiv.org/abs/2402.03038

[23] Nagy E, Janisch M, Hržić F, Sorantin E, Tschauner S (2022) A pediatric wrist trauma x-ray dataset (GRAZPEDWRI-DX) for machine learning. Sci Data 9. 10.1038/s41597-022-01328-z 10.1038/s41597-022-01328-z PMC9122976 35595759

[24] Raza R, Zulfiqar F, Khan MO et al (2023) Lung-effnet: lung cancer classification using efficientnet from CT-scan images. Eng Appl Artif Intell 126:106902. 10.1016/j.engappai.2023.106902

[25] Ali K, Shaikh ZA, Khan AA, Laghari AA (2022) Multiclass skin cancer classification using efficientnets–a first step towards preventing skin cancer. Neurosci Inform 2:100034. 10.1016/j.neuri.2021.100034

[26] Latha M, Kumar PS, Chandrika RR, Mahesh T, Kumar VV, Guluwadi S (2024) Revolutionizing breast ultrasound diagnostics with efficientnet-b7 and explainable ai. BMC Med Imaging 24:230 39223507 10.1186/s12880-024-01404-3 PMC11367906

[27] Tan M, Le Q (2019) Efficientnet: Rethinking model scaling for convolutional neural networks. In International Conference on Machine Learning. pp. 6105–6114. PMLR.

[28] Howard J et al (2018) fastai. GitHub

[29] Ju R-Y, Chien C-T, Lin C-M, Chiang J-S (2024) Global context modeling in YOLOv8 for pediatric wrist fracture detection. International Symposium on Intelligent Signal Processing and Communication Systems (ISPACS), Kaohsiung, Taiwan, pp. 1–5. 10.1109/ISPACS62486.2024.10869064

[30] Jocher G, Chaurasia A, Qiu J (2024) Ultralytics YOLO. https://github.com/ultralytics/ultralytics

[31] Jocher G et al (2024) Ultralytics: YOLO Performance Metrics—docs.ultralytics.com. https://docs.ultralytics.com/guides/yolo-performance-metrics/#introduction. Accessed 29-Aug-2024

[32] Tian J, Jin Q, Wang Y, Yang J, Zhang S, Sun D (2024) Performance analysis of deep learning-based object detection algorithms on coco benchmark: a comparative study. J Eng Appl Sci 71. 10.1186/s44147-024-00411-z

[33] Rezatofighi H, Tsoi N, Gwak J, Sadeghian A, Reid I, Savarese S (2019) Generalized intersection over union: a metric and a loss for bounding box regression. In: Proceedings of the IEEE/CVF Conference on Computer Vision and Pattern Recognition (CVPR). Long Beach, CA, USA: IEEE. pp. 658–666. 10.1109/CVPR.2019.00075

[34] Altmann-Schneider I, Kellenberger CJ, Pistorius S-M et al (2023) Artificial intelligence-based detection of paediatric appendicular skeletal fractures: performance and limitations for common fracture types and locations. Pediatr Radiol 54:136–145. 10.1007/s00247-023-05822-3 38099929 10.1007/s00247-023-05822-3 PMC10776701

[35] Karimi-Rouzbahani H, Bagheri N, Ebrahimpour R (2017) Invariant object recognition is a personalized selection of invariant features in humans, not simply explained by hierarchical feed-forward vision models. Sci Rep 7. 10.1038/s41598-017-13756-8 10.1038/s41598-017-13756-8 PMC5663844 29089520

